# Study on the spatial specificity of phenolics in fruit of different jujube varieties

**DOI:** 10.1038/s41598-023-46228-3

**Published:** 2023-11-02

**Authors:** Xiaofeng Zhou, Zhun Zhang, Yunfeng Pu, Cuiyun Wu, Min Yan, Qiaoqiao Zhang

**Affiliations:** 1 College of Life Science and Technology, Tarim University, Alar, 843300 Xinjiang China; 2 College of Horticulture and Forestry, Tarim University, Alar, 843300 Xinjiang China; 3 College of Food Science, Tarim University, Alar, 843300 Xinjiang China; 4 The National-Local Joint Engineering Laboratory of High Effciency and Superior-Quality Cultivation and Fruit Deep Processing Technology on Characteristic Fruit Trees, Alar, 843300 Xinjiang China; 5 Xinjiang Production and Construction Corps Key Laboratory of Protection and Utilization of Biological Resources in Tarim Basin, Alar, 843300 Xinjiang China

**Keywords:** Plant sciences, Plant physiology

## Abstract

Phenol, an important bioactive substance in jujube fruit, is one of the most important indexes of fruit quality. In order to clarify the phenolic substance composition, content and distribution characteristics of different varieties of jujube fruits, this study measured and compared the content of total phenols, total flavonoids and phenolic substance composition in different parts of fruits of six jujube varieties, and combined with the principal component analysis, the results showed that: Fourteen phenolic substances were identified in the fruits of different jujube varieties, and proanthocyanidins, epicatechin, catechin and rutin were the main components, accounting for 58.60%, 16.08%, 13.56% and 5.57% of the total phenolic substances, respectively. The total phenolic and total flavonoid contents of jujube fruits of each variety were between 18.11 and 21.45 mg GAE/g DW and 10.56–20.25 mg RE/g DW, respectively, and the differences in the contents between the varieties were significant, and the contents of ‘Junzao’ and ‘Jinlingyuanzao’ were higher than those of other varieties.The phenolic content of different parts of jujube fruits showed spatial distribution differences, manifested as the total phenols, total flavonoids and their fractions of the peel were significantly higher than that of the pulp, while the content of the pulp near the kernel was significantly higher than that of other parts of the pulp, This study provides a theoretical basis for analysing the differences in phenolic substances in jujube fruits.

## Introduction

Jujube (Ziziphus jujuba Mill.), a genus of jujube in the family Rhamnaceae, is one of the advantageous fruit tree species with unique characteristics and high economic and medicinal values in China^1^, as a medicinal and food plants, it is rich in various bioactive substances, which have the effect of liver protection, enhancement of antioxidant enzyme activity in the body, prevention of cardiac muscle damage and oxidative damage, etc^2–4^. Therefore, as people increasingly pay attention to health preservation and the pursuit of fruit health care, jujube has become one of the fastest growing fruit tree species in China in recent years. Especially, the jujube industry in Xinjiang has developed rapidly, and its output has increased by 86.8% from 2010 to 2020^5, 6^. In recent years, it has also become increasingly popular in the international market and exported to Southeast Asia, East Asia and Europe. Moreover, the regional conditions of Xinjiang have bred more abundant fruit nutrition and functional components^7, 8^.

It has been shown that phenolics are one of the most important functional components of jujube fruit, and their content varies considerably in different varieties^9^. Xue^10^ studied 245 jujube germplasm and found that the total flavonoid content was widely distributed (0.25 mg/g FW ~ 2.77 mg/g FW), and the difference between high flavonoid germplasm and low flavonoid germplasm was 11 times. The study on 10 jujube varieties in Xinjiang showed^11^ that the content of total flavonoids in fruits of ‘Huizaoyouzhu’ and ‘Zan2’ was significantly higher than that of other varieties. At the same time, the study further found that there were significant differences in the composition and content of phenolic substances among varieties. Shi^12^ detected four substances, myricetin, rutin, quercetin and isorhamnetin, in ‘Jinsixiaozao’. Yang^13^ detected five substances, i.e. Vitexin, isoquercetin, quercetin, porphyritin and isorhamnetin, from ‘Guangzao’. Zou^14^ detected six substances including gallic acid, protocatechuic acid, catechin, chlorogenic acid, ferulic acid and rutin in ‘Yuanlingdazao’. Among the eight representative jujube varieties planted in Shanxi, the contents of total phenol, betulinic acid and oleanolic acid in fruit of ‘Jishanbanzao’ are higher than those of other varieties^15^. Among the 8 jujube varieties determined by Di et al.^16^, the rutin content of ‘Shanbeiyuanhongzao’ was the highest (288.21 μg/g).

Jujube fruits show differences in phenolics due to different parts of the fruit, and the results of a large number of studies have shown that the total phenol and total flavonoid contents of jujube peel are much higher than those of the pulp and kernel^17–19^. Zhang et al.^20^ detected chlorogenic acid, gallic acid, protocatechuic acid and caffeic acid in the peel, pulp and seeds of 'Dongzao', 'Muzao', and 'Hamidazao'. and they were mainly distributed in the peel, followed by the pulp. Siriamornpun et al.^21^ detected a total of seven phenolic acids in different parts of the jujube fruits, including gallic acid, protocatechuic acid, parahydroxybenzoic acid, P-coumaric acid, ferulic acid, butyric acid, and erucic acid, among which The content of gallic acid and protocatechuic acid in the seed was higher than that in the pulp. Wang et al.^22^, in testing the distribution and content of different types of phenolic acids in various parts of jujube fruits, found that gallic acid, protocatechuic acid and P-hydroxybenzoic acid, caffeic acid, P-coumaric acid, ferulic acid, cinnamic acid, and chlorogenic acid were found; and that they were mainly distributed in the peel, and the phenolic acids in the peel and the seed existed mainly in the insoluble bond state, and phenolic acids in the pulp are mainly present in the glycosidic state.

Phenolic substances are important indicators of fruit quality and an important factors affecting fruit flavour^23^. In actual production, the quality of the same jujube variety in different years and different cultivation modes has obvious differences, but the substances that cause the differences in their fruits need to be further studied. Therefore, in order to better understand the distribution of phenolic substances in jujube fruits, this study takes Xinjiang's main cultivar of jujube and other cultivars as materials, and focuses on comparing the differences in the composition and content of total flavonoids, total phenols, and phenolic substances in different jujube cultivars and different parts of the fruits, with a view to providing a certain theoretical basis and reference for the regulation of quality and genetic improvement of jujube.

## Results

### Comparison of differences in phenolic contents of the fruits of different jujube varieties

#### Analysis of differences in total phenolic and total flavonoid contents of the fruits of different jujube varieties

**Figure 1:**
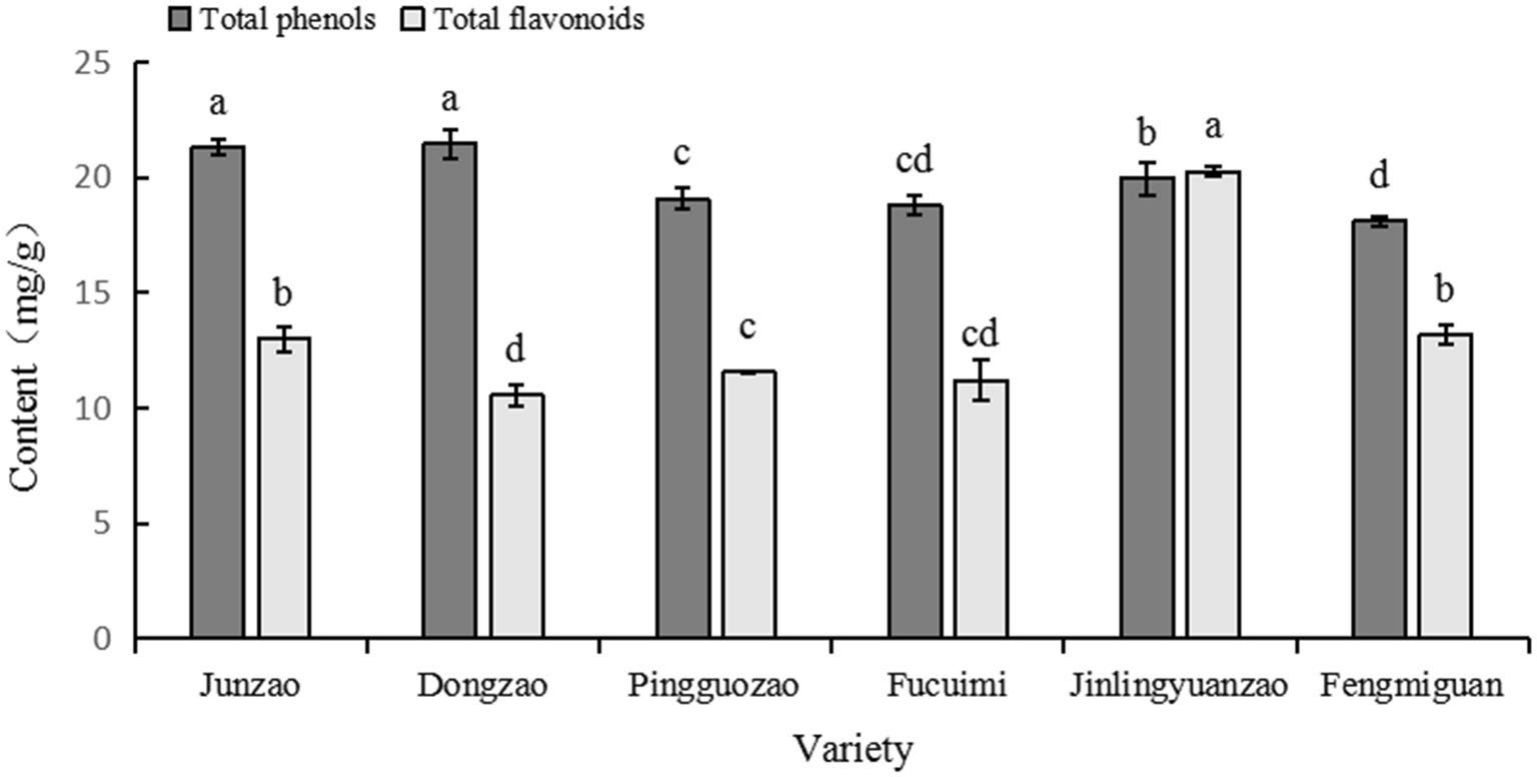
Comparison of total phenolic content (mg GAE/g DW) and total flavonoids content (mg RE/g DW) of the fruits of different jujube varieties.

It can be seen from Fig. 1 that the content distribution ranges of total phenols and total flavonoids in the fruits of six jujube varieties are 18.11–21.45mg GAE/g DW and 10.56–20.25mg RE/g DW, respectively. Except for ‘Jinlingyuanzao’, the total phenolic contents of the fruits of the remaining five jujube varieties were higher than the total flavonoid contents. The total phenolic content of the ‘Jinlingyuanzao’ frui was significantly lower than that of ‘Junzao’ and ‘Dongzao’, and significantly higher than that of ‘Pingguozao’, ‘Fucuimi’ and ‘Fengmiguan’. The total phenolic content of the ‘Pingguozao’ fruit was significantly higher than that of ‘Fengmiguan’ and was not significantly different from that of ‘Fucuimi’. There was no significant difference in the total phenolic contents between ‘Junzao’ and ‘Dongzao’ (*P*<0.05). The content of total flavonoids in the fruit of ‘Jinlingyuanzao’ was significantly higher than that of the other jujube varieties. There was no significant difference in the total flavonoid contents between ‘Junzao’ and ‘Fengmiguan’ (*P*<0.05), and the total flavonoid contents of the fruits of ‘Junzao’ and ‘Fengmiguan’ were significantly higher than those of ‘Dongzao’, ‘Pingguozao’ and ‘Fucuimi’, while the total flavonoid content in the fruit of ‘Pingguozao’ was significantly higher than that of ‘Dongzao’ and was not significantly different from that of ‘Fucuimi’. On the whole, ‘Junzao’ and ‘Jinlingyuanzao’ were superior to the other four jujube varieties in phenolics, and the patterns of total phenolic and total flavonoid contents of the fruits of the two were very different.

#### *Analysis of the *differences* in the contents of phenolic components in the fruits of different jujube varieties*

**Figure 2:**
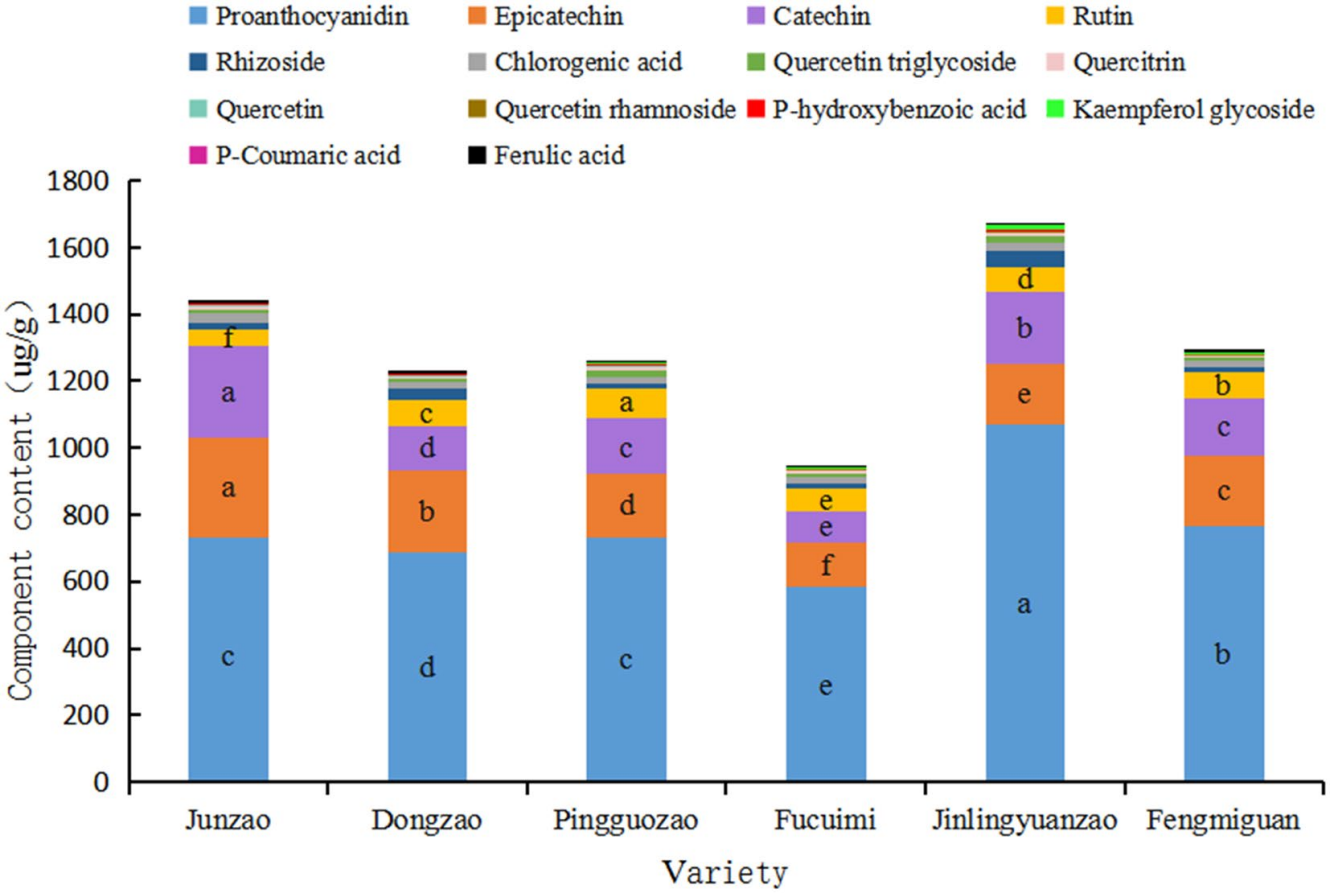
Comparison of phenolic component contents of different jujube varieties.

It can be seen from Figure 2 that 14 kinds of phenolic substances, including proanthocyanidins, epicatechin, catechins, rhizobioside, rutin, chlorogenic acid, quercetin, quercitrin, P-hydroxybenzoic acid, quercetin triglycoside kaempferol glycoside, ferulic acid, quercetin rhamnoside and P-coumaric acid, were detected in the fruits of the six jujube varieties tested. The main components were proanthocyanidin, epicatechin, catechin and rutin, accounting for 58.60%, 16.08%, 13.56% and 5.57% of the total phenolics, respectively. From the content of the main components mentioned above, proanthocyanidins, epicatechin, catechin and rutin contents were significantly different among the varieties. The proanthocyanidin content of the fruit showed: ‘Jinlingyuanzao’ > ‘Fengmiguan’ > ‘Junzao’ and ‘Pingguozao’ > ‘Dongzao’ > ‘Fucuimi’, Epicatechin content: ‘Junzao’ > ‘Dongzao’ > ‘Fengmiguan’ > ‘Pingguozao’ > ‘Jinlingyuanzao’ > ‘Fucuimi’, Catechin content: ‘Junzao’ > ‘Jinlingyuanzao’ > ‘Fengmiguan’ and ‘Pingguozao’ > ‘Fucuimi’, Rutin content: ‘Pingguozao’ > ‘Fengmiguan’ > ‘Dongzao’ > ‘Jinlingyuanzao’ > ‘Fucuimi’ > ‘Junzao’.

### Comparison of differences in phenolic contents of different parts of jujube fruits

#### *Analysis of *the* differences in total phenolic and total flavonoid contents of different parts of jujube fruits*

**Figure 3:**
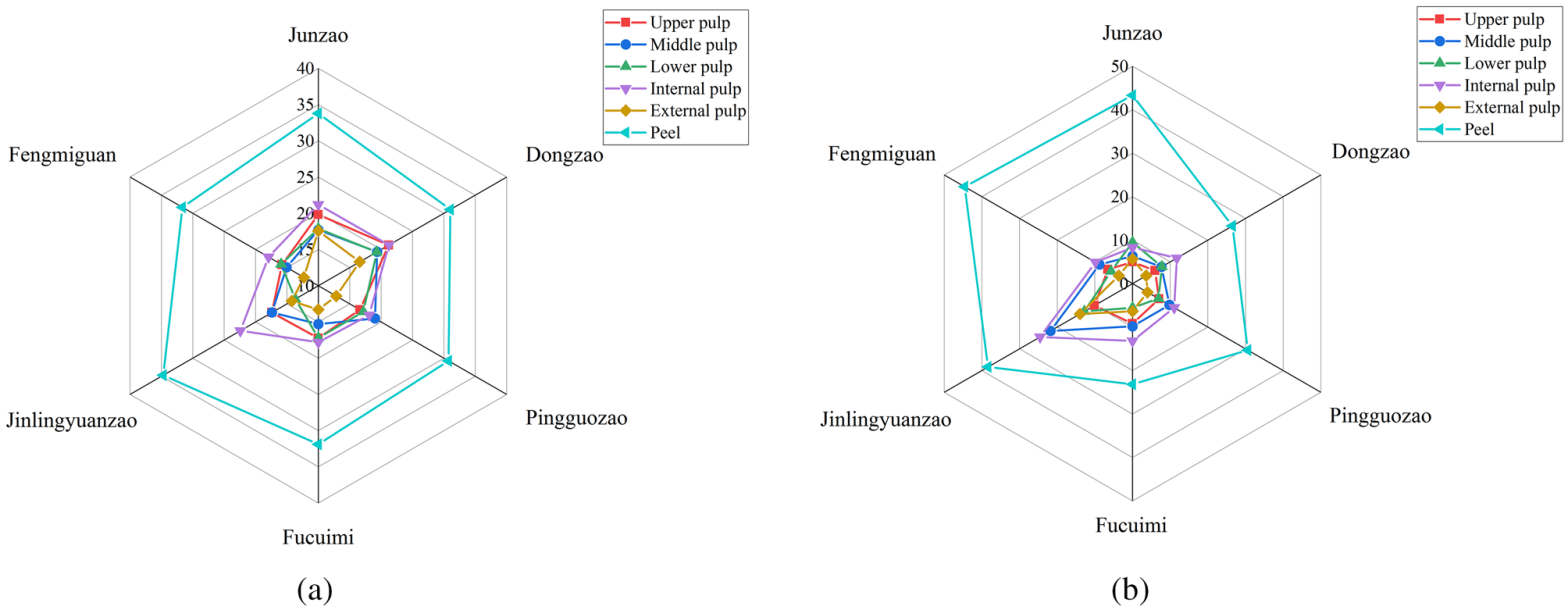
Comparison of total phenolic (a mg GAE/g DW) and flavonoid (b mg RE/g DW) contents of different parts of jujube fruits.

It can be seen from Fig. 3 that the contents of total phenols and total flavonoids in different parts of the fruits of the six jujube varieties ranks as follows: peel>pulp (upper, middle, lower, internal and external). Among them, the average contents of total phenols in the peel and pulp of the six jujube varieties were 32.30 mg GAE/g DW and 17.29 mg GAE/g DW, respectively, and the total phenolic content in the peel was 1.87 times higher than that in the pulp. The average contents of total flavonoids in the peel and pulp of the six jujube varieties were 34.43 mg RE/g DW and 9.07 mg RE/g DW, respectively, with a difference of 3.80 times. The radar chart shows that the total phenolic and total flavonoid contents of the external pulp are closest to the radar center, indicating that the contents of total phenols and total flavonoids in the external pulp of the six jujube varieties are the lowest, with averages of 14.47 mg GAE/g DW and 6.20 RE/g DW, respectively. On the contrary, the contents of total phenols and total flavonoids in the internal pulp were higher, with average values of 19.78 mg GAE/g DW and 13.11 mg RE/g DW, respectively. From the horizontal direction, it can be seen that the total phenolic and total flavonoid contents of the pulp in jujube fruits show a horizontal direction of decreasing from the inside to the outside. The vertical distribution characteristics of total phenol and total flavonoid contents in the pulp of each jujube variety were different.

#### Analysis of the composition and content differences in phenolic substances in different parts of jujube fruits

**Figure 4:**
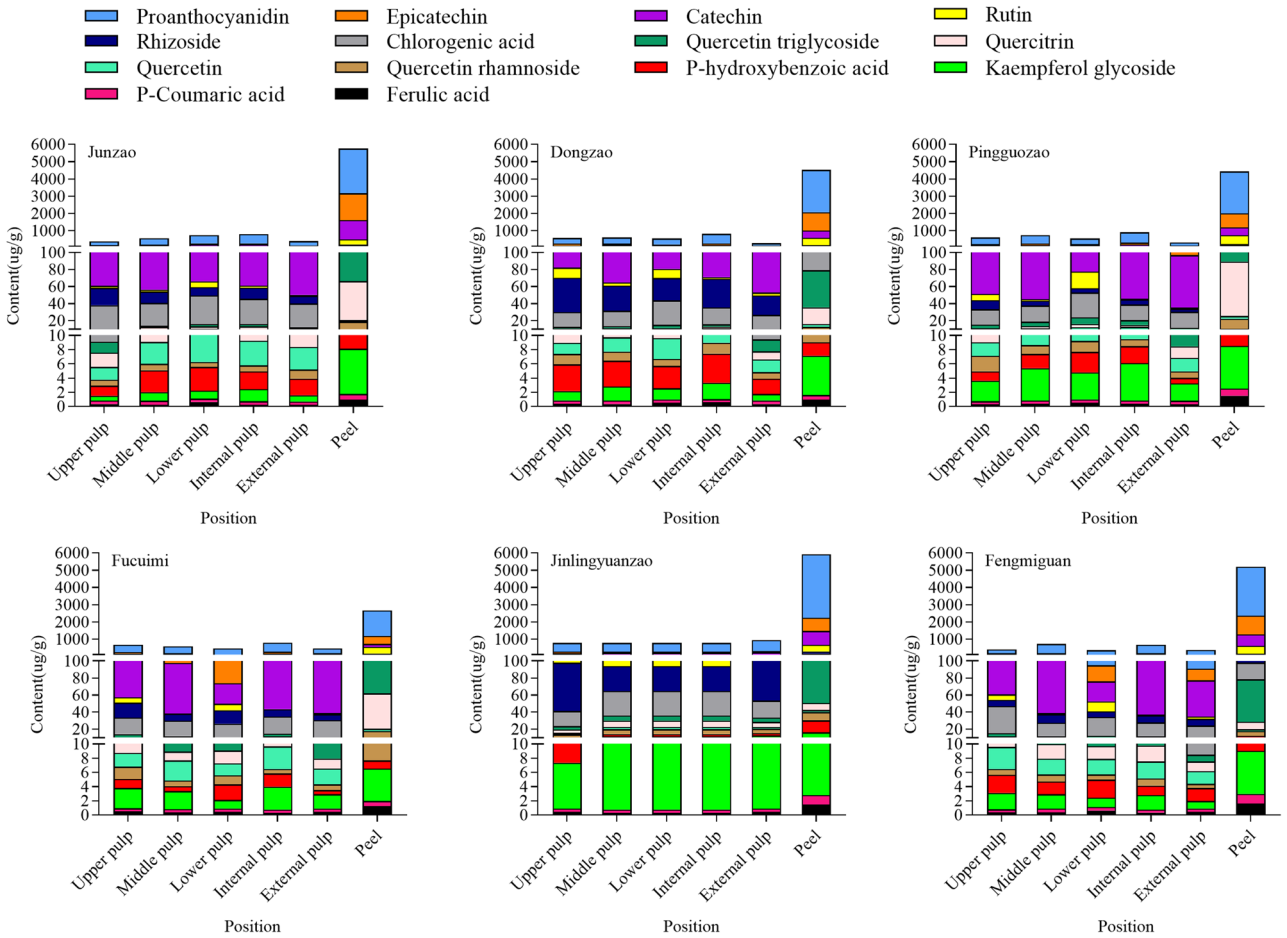
Comparison of phenolic compositions and contents of different parts of 6 red jujube cultivars. Unit: µ g/g DW.

As shown in Fig. 4, 14 phenolic substances were detected in all parts of the jujube fruit, with proanthocyanidins, catechins, epicatechin, chlorogenic acid, rhizobioside and rutin being the main phenolic substances, and all of the tested jujube fruit varieties showed higher phenolic content in the peel than in the pulp (upper, middle, lower, internal and external). The distribution of the content of proanthocyanidins, epicatechin and rhizobioside in the main compositions of different parts of the pulp were shown: the content of the internal pulp of the fruit was the highest, the content of the external pulp was the lowest, and the content decreased horizontally from the pulp near the kernel of the jujube to the pulp near the peel at the equator(except for ‘Jinlingyuanzao’ and ‘Fengmiguan’). These results show that the distribution of polyphenols in the whole jujube fruit is uneven, and that the key reason for this is due to differences in the content of the major components.

### Principal component analysis

**Figure 5:**
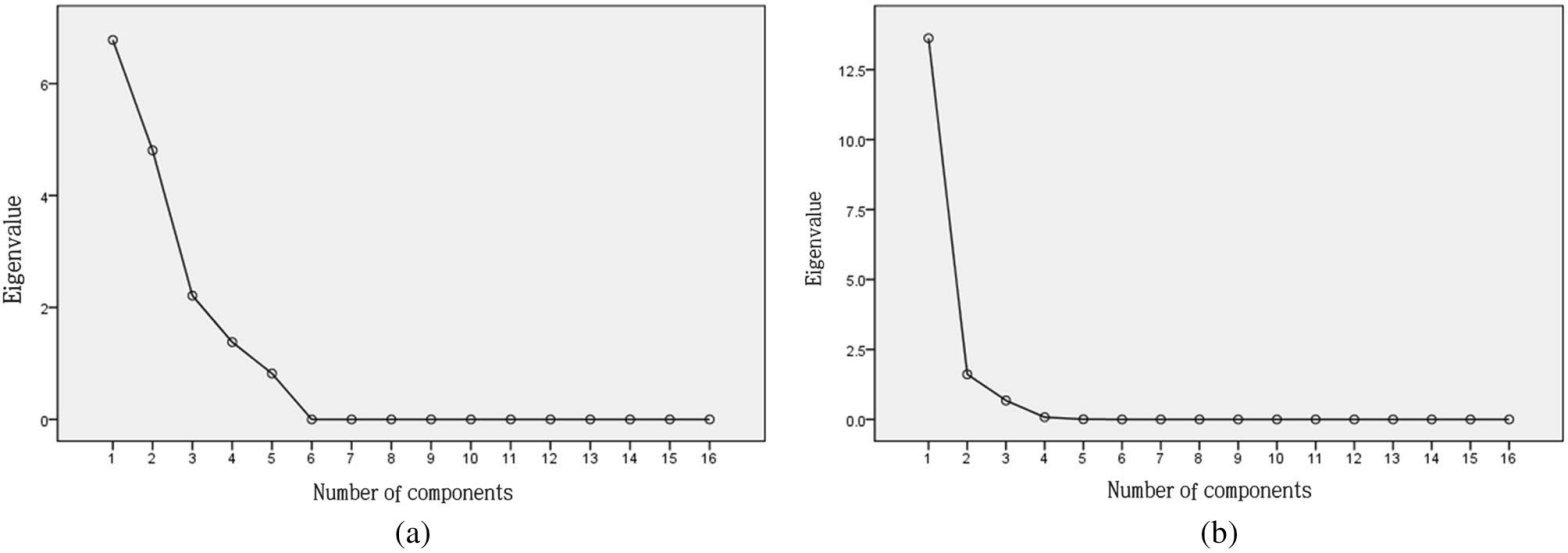
Gravel map of total phenols, total flavonoids and 14 phenolic substances in different jujube varieties (**a**) and different parts of jujube fruits (**b**).

The principal component analysis of total phenols, total flavonoids and 14 phenolic substances in different parts of the fruits of different jujube varieties (Figure 5) shows that when the abscissa is 4 (a) and 2 (b), the broken line suddenly becomes more stable. Based on this, it can be concluded that the first four major components can effectively measure the content distribution of phenolic substances in the fruits of different jujube varieties; however, only the first two main components can effectively compare the content differences in phenolic substances between different parts of the fruit.

According to the extraction results of the main components of phenolic substances in different parts of the fruits of different jujube varieties (Table 1), further analysis shows that the first main component of phenolic substances in the fruits of jujube varieties is represented by proanthocyanidins and total flavonoids, the second main component is represented by ferulic acid and epicatechin, the third main component is represented by quercetin and quercitrin, and the fourth main component is represented by P-coumaric acid. Since the cumulative contribution rate of variance of the first four main components reached 94.87%, the above seven substances can effectively reflect the distribution of phenolic substances in the fruits of different jujube varieties. The first principal component of phenolic substances in different parts of jujube fruit is represented by rutin, quercetin triglycoside and proanthocyanidins, and the second principal component is represented by chlorogenic acid and quercetin. The cumulative contribution rate of variance of the first two major components is 95.24%, indicating that the above four substances can effectively respond to the distribution of phenolic substances in different parts of jujube fruit.

**Table 1:** Extraction results of main components of phenolic substances in different parts of the fruits of different jujube varieties.

Phenolic substances	Components of different varieties				Components of different position	
	1	2	3	4	1	2
Proanthocyanidin	0.941	−0.288	−0.061	0.163	0.998	−0.039
Epicatechin	0.124	0.781	−0.305	0.441	0.974	−0.089
Catechin	0.687	0.574	0.114	0.431	0.953	−0.072
Rutin	−0.213	−0.760	0.087	0.173	0.999	−0.081
Chlorogenic acid	0.611	0.751	0.135	0.112	0.142	0.804
Rhizobioside	0.794	−0.088	−0.512	−0.314	0.950	−0.208
Quercetin triglycoside	0.721	−0.409	0.470	−0.131	0.999	−0.073
Quercitrin	0.049	0.393	0.840	−0.275	0.984	−0.054
Quercetin	0.256	0.740	0.619	0.052	0.214	0.871
Quercetin rhamnoside	0.856	−0.321	0.256	−0.305	0.996	−0.074
P−hydroxybenzoic acid	0.869	−0.122	−0.451	−0.037	0.889	0.342
Kaempferol glycoside	0.866	−0.441	0.104	−0.207	0.952	0.069
P-coumaric acid	0.650	−0.359	0.133	0.656	0.996	−0.081
Ferulic acid	−0.395	−0.866	0.101	0.277	0.985	0.015
Total phenols	0.306	0.709	−0.443	−0.300	0.945	0.033
Total flavonoids	0.927	−0.266	−0.007	0.060	0.963	0.025
Eigenvalue	6.780	4.806	2.211	1.381	13.624	1.613
Percentage of variance %	42.378	30.040	13.82	8.633	85.153	10.083
Cumulative percentage %	42.378	72.418	86.238	94.871	85.153	95.235

According to the percentage of characteristic values, a comprehensive scoring model is built:

- Comprehensive score of different jujube varieties = 42.378% × Y(i,1) + 30.040% × Y(i,2) + 13.82% × Y(i,3) + 8.633% × Y(i,4);
- Comprehensive score of different parts of jujube fruit = 85.153% × Z(i,2) + 10.083% × Z(i,2);
- According to the above model, the comprehensive scores of phenolic substances in different jujube varieties and different parts of the fruit are shown in Tables 2 and 3.

**Table 2:** Comprehensive score of phenolic substances in the fruits of different jujube varieties.

Variety	Y1	Y2	Y3	Y4	Composite score	Sort
Junzao	0.88	4.20	0.29	0.53	1.72	1
Dongzao	−1.18	0.34	−2.29	−0.71	−0.78	4
Pingguozao	−0.62	−0.67	2.23	0.06	−0.15	3
Fuicuimi	−2.54	−0.57	0.57	−1.41	−1.29	6
Jinlingyuanzao	4.81	−1.56	−0.20	−0.45	1.50	2
Fengmiguan	−1.35	−1.74	−0.61	1.98	−1.01	5

**Table 3:** Comprehensive score of phenolic substances in different parts of jujube fruit.

Posiyion	Z1	Z2	Composite score	Sort
Upper pulp	−1.65	−1.05	−1.51	5
Middle pulp	−1.43	−0.06	−1.22	4
Lower pulp	−1.21	1.95	−0.84	3
Internal pulp	−1.03	0.91	−0.78	2
External pulp	−2.17	−1.51	−2.00	6
Peel	7.49	−0.24	6.35	1

**Figure 6:**
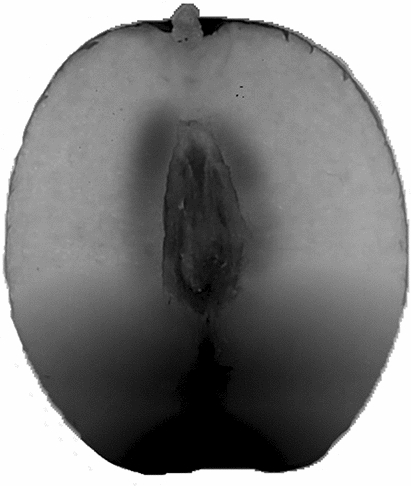
Prediction of phenolic content distribution in different parts of jujube fruit. * The darker the simulated color, the higher the content of phenolic substances.

It can be seen from Table 2 that the six jujube varieties are ranked as ‘Junzao’, ‘Jinlingyuanzao’, ‘Pingguozao’, ‘Dongzao’, ‘Fengmiguan’ and ‘Fucuimi’. Therefore, among the tested jujube varieties, ‘Junzao’ and ‘Jinlingyuanzao’ can be given priority as a health food. It can be seen from Table 3 that the peel has the highest content of phenolics, and the distribution of phenolic substances in different parts of the jujube pulp is ranked as internal>lower>middle>upper>external. Therefore, the results further support that the phenolic content of the external pulp is the lowest, and the predicted phenolic content of the jujube fruit is higher in the areas closest to the core and in the lower pulp (as shown in Figure 6).

## Discussion

The Phenolic substances are one of the most important and widely distributed secondary metabolites in fruit^24^ and a major factor in the formation of antioxidant capacity of fruits^25^, and their fractions and contents vary significantly among various fruit tree species^26–30^.Previous studies have shown that the main phenolics of the fruit among jujube varieties are epicatechin, catechin and rutin^31^. In contrast, 14 phenolic substances were detected in the fruits of the 6 jujube varieties tested in this study. The main phenolic substances were proanthocyanidins, followed by epicatechin, catechin and rutin, the content of proanthocyanidins was significantly higher than that of other phenolic substances. In addition, Chang^32^ detected a total of eight phenolics in ‘Goutouzao’ fruits, and in addition to rutin and catechin, chlorogenic acid was also the main phenolics. Zhu^33^ et al. showed that the nutrient differences in different jujube fruit of the same variety may be caused by different cultivation environments and management measures. In this study, the six jujube varieties tested were all collected from the same place, and the cultivation environment and management measures were consistent, but the total phenol and total flavonoid contents of ‘Junzao’ and ‘Jinlingyuanzao’ were higher than those of the remaining four varieties of jujube, indicating that the differences in total phenolic and total flavonoid contents of jujube fruits among varieties are related to the genotype of jujube.

The compositions and contents of polyphenols in different tissues of the same plant are also different. The total phenolic contents of pears^34^, grapes^28^, oranges^30^, peaches^35^, pomegranates^36^, kiwifruit^37^ and other fruits are in the form of peel>pulp, of which the total phenolic content of kiwifruit peel is about 8–16 times that of pulp. In this study, the contents of phenolic substances in jujube fruit are also shown as peel>pulp, which is consistent with the research results of Wu Benpei^18^ and Liu Cong^19^, in which the contents of total phenols and total flavonoids in peel is 1.87 times and 3.80 times higher than that in pulp, respectively. By analysing the cellular ultrastructure of ripening fruits, it was found that most of the organelles in the peel and pulp were disassembled, but the endoplasmic reticulum was still abundant in the peel^38^, and the structure of mitochondria in the pulp still remained intact^39, 40^. It was hypothesized that since the abundance of endoplasmic reticulum in the peel was favourable to the synthesis of organic matter, and the continuous integrity of mitochondria in the pulp was able to oxidatively decompose the organic matter, it was concluded that the endoplasmic reticulum and the mitochondria were the key factors contributing to the superiority of the peel's phenolics compared to that of the pulp.

Phenolics are important bioactive substances and nutrients in fruit fruits, and their composition and abundance are key indicators characterising the quality of the fruit, as well as important factors determining the taste^41–43^. Previous studies have found that phenolic compounds are related to the astringent, bitter and other flavours of fruits^23^, which is consistent with the acidic and astringent flavour of the pulp at the kernel of the near jujube fruit in this study. The contents of phenolic substances in different parts of jujube flesh were comprehensively evaluated. The distribution of phenolic substances was ranked as internal>lower>middle>upper>external, which indicated that the contents of phenolic substances in the whole jujube fruit were uneven, in which the closer to the internal pulp and the lower pulp, the higher the content, it is assumed that the synthesis and transport capacity of phenolics the internal pulp and the lower pulp is stronger than the rest of the parts, which provides a complementary and reference for the quality regulation and genetic improvement of jujube.

## Methods

### Chemicals

All reagents, HPLC-grade phenolic standards (proanthocyanidins, catechins, epicatechin, chlorogenic acid, rutin, rhizobioside, quercetin, quercitrin, P-hydroxybenzoic acid, quercetin triglycoside kaempferol glycoside, ferulic acid, quercetin rhamnoside and P-coumaric acid) were also purchased from Sigma–Aldrich (St. Louis, MO, USA). Methanol, acetonitrile and formic acid were purchased from Tedia Company, Inc. (Fairfield, CT, USA). Folin-Ciocalteu reagent and all other chemicals were obtained from Sinopharm Chemical Reagent Co. (Shanghai, China).

### Sample collection

Fruits were collected from the jujube germplasm resources nursery of Tarim University (40° 32′ 23.50″ N, 81° 17′ 41.61″ E), Alar, Xinjiang, China. In this experiment,permission for sample collection was granted by Tarim University. Jujube plants of the same age, structure, and consistent management level were selected, and three trees were sampled for each cultivar. All methods involving plants were carried out in accordance with relevant national, international, or institutional guidelines and regulations.

**Figure 7:**
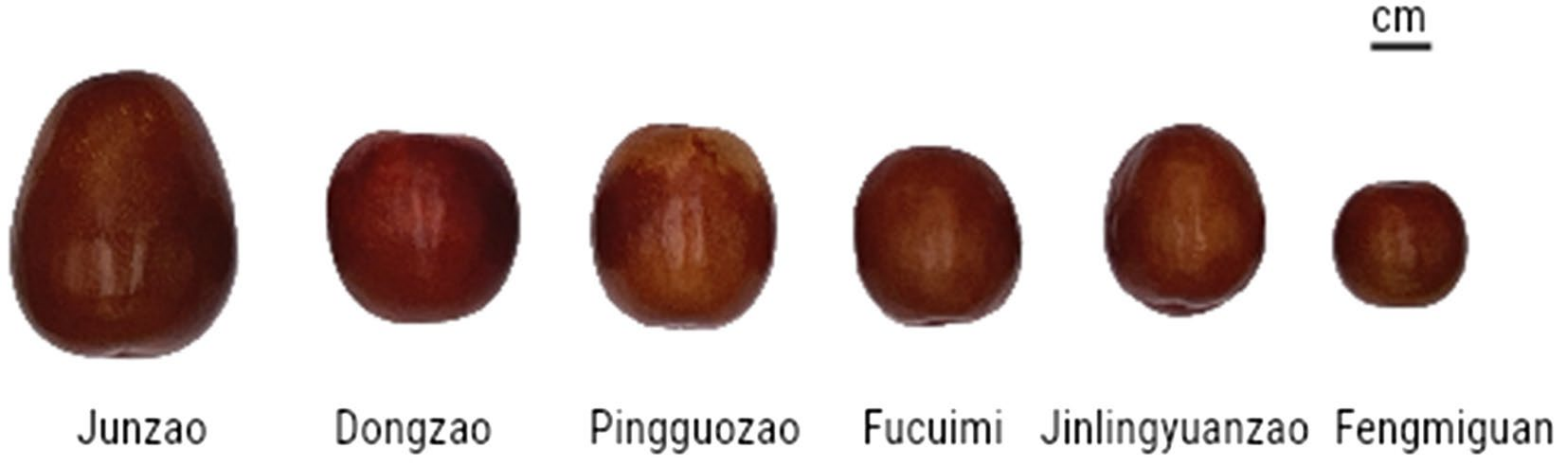
Picture of 6 jujube fruits.

**Figure 8:**
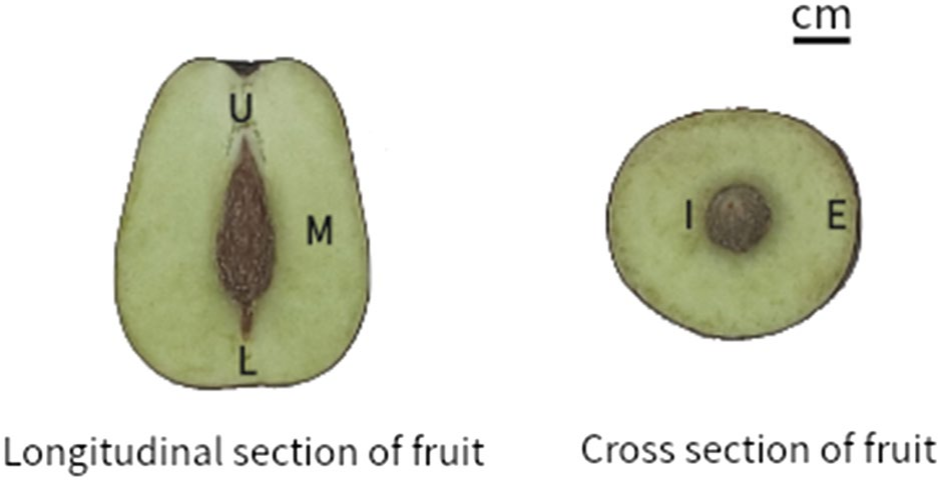
Schematic diagram of jujube fruit sampling. U: the upper pulp (peeled pulp at the shoulder), M: the middle pulp (peeled pulp at the middle of the fruit), L: the lower pulp (peeled pulp at the top of the fruit), I: the internal pulp (0.2 ~ 0.5 cm outside the core), E: the external pulp of the fruit (about 0.2 ~ 0.5 cm under the peel).

A total of six cultivars of jujuba were investigated, including ‘Junzao’, ‘Dongzao’, ‘Pingguozao’, ‘Fucuimi’, ‘Jinlingyuanzao’ and ‘Fengmiguan’. Jujube fruits were collected from September to October 2020 at the crisp and ripe stage (Figure 7). Each variety randomly picked 20 jujube fruits from different parts of each tree, and three biological replicates, with a total of 60 fruits from three trees, none of which had any obvious spots or diseases. The fresh fruit were treated immediately after the fruit had been transported to the laboratory, The fruits were separated and sampled under low temperature according to the jujube peel, the external pulp of the fruit (about 0.2–0.5cm under the peel), the internal pulp (0.2–0.5cm outside the core), the upper pulp (peeled pulp at the shoulder), the middle pulp (peeled pulp at the middle of the fruit), and the lower pulp (peeled pulp at the top of the fruit) (Figure 8). A mixture of 20 samples of the same part of the fruit from the same tree, and three biological replicates. The sliced fruit was then frozen using a freeze dryer (The FreeZone 6, Labconco, USA). Lyophilized samples were homogenized in a domestic blender (JYL-C022E, Jiuyang, China), and the powder was packed in airtight polyethylene bags. Finally, the powder was stored at -80 °C before further analysis.

### Preparation of extract

The 1.0 g freeze-dried jujube powder was extracted with 25 mL of aqueous methanol (80%, v/v) under ultrasound at 300 W for 20 min. The extracts were used for the determination of total phenols, total flavones, and polyphenols.

### Determination of total polyphenol content and total flavonoid content

To determine the total phenolic and total flavonoid contents of jujube fruit, we referred to the method of Xi Jiang^44^ and slightly modified it. Total flavonoids were determined at 510 nm and total phenols at 760 nm, and the linear regression equations were established by using gallic acid (mg GAE/g DW) and rutin (mg RE/g DW) as the standards, respectively. As shown in Table 4.

**Table 4:** Standard curve equation of total phenols and total flavonoids.

Substance	Linear regression equation	R^2^
Total phenols	y = 7.182x + 0.017	0.999
Total flavonoids	y = 0.323x + 0.001	0.999

### Determination of phenolic profiling

The phenolic profile was produced according to the method described previously by Pu^45^, with some modification. We used a ZORBAX SB-C18 (250 × 4.6 mm, id) chromatographic column (Agilent, USA). A 0.45 µ m microfiltration membrane was used to filter the extract into a 1.5 mL sample injection bottle. Then, we used high-performance liquid chromatography to determine phenolic substances. The mobile phase was 0.5% formic acid (eluent B) and methanol (eluent A). The column oven temperature was set to 30 ℃. The flow rate of the solvent system is 0.7 mL/min, and the injection volume is 10 μ L.The gradient program of the samples are shown in Table 5. The phenol extraction time and wavelength are shown in Table 6. The phenolic substance content in the sample is expressed in µg/gDW.

**Table 5:** Gradient program of the sample.

Time	A	B
0 ~ 6 min	10%	90%
6 ~ 10 min	10 ~ 20%	90 ~ 80%
10 ~ 11 min	20 ~ 25%	80 ~ 75%
11 ~ 15 min	25 ~ 30%	75% ~ 70%
15 ~ 25 min	30 ~ 40%	70 ~ 60%
25 ~ 32 min	40 ~ 55%	60 ~ 45%
32 ~ 40 min	55 ~ 100%	45 ~ 0%
40 ~ 50 min	100%	0%
50 ~ 51 min	100 ~ 10%	0 ~ 90%B
51 ~ 60 min	10%	90%

**Table 6:** Determination method of phenols.

Wavelength nm	Phenol extraction time min	Phenolic substances
254	22.616	P-hydroxybenzoic acid
278	24.659	Proanthocyanidin
278	21.244	Catechin
278	25.632	Epicatechin
284	38.234	Rhizoside
310	31.612	P-coumaric acid
324	33.331	Ferulic acid
326	23.002	Chlorogenic acid
355	32.203	Quercetin triglycoside
355	35.584	Kaempferol glycoside
355	36.888	Rutin
355	38.956	Quercitrin
355	39.484	Quercetin rhamnoside
370	41.944	Quercetin

### Statistical analysis

SPSS version 17.0 (SPSS Inc., Chicago, IL, USA) was used to perform statistical analysis of the collected data. The data were analyzed using one-way analysis of variance (ANOVA), and Duncan’s test was performed to determine correlations at the p<0.05 significance level. Furthermore, principal composition analysis (PCA) was performed using Origin 2019 software for charting.

### Supplementary Information

Supplementary Information.

## Data Availability

All data generated or analysed during this study are included in this published article [and its supplementary information files].
